# G_0_-PCC-FISH derived multi-parametric biodosimetry methodology for accidental high dose and partial body exposures

**DOI:** 10.1038/s41598-024-65330-8

**Published:** 2024-07-12

**Authors:** Usha Yadav, Nagesh N. Bhat, Utkarsha S. Mungse, Kapil B. Shirsath, Manish Joshi, Balvinder K. Sapra

**Affiliations:** 1 Radiological Physics and Advisory Division, Bhabha Atomic Research Centre, Mumbai, 400085 India; 2 Homi Bhabha National Institute, Anushaktinagar, Mumbai, 400094 India

**Keywords:** G_0_-PCC-FISH, Partial body exposure, Premature chromosome condensation, High dose biodosimetry, Whole chromosome painting, Biological techniques, Biophysics, Biotechnology, Cell biology

## Abstract

High dose radiation exposures are rare. However, medical management of such incidents is crucial due to mortality and tissue injury risks. Rapid radiation biodosimetry of high dose accidental exposures is highly challenging, considering that they usually involve non uniform fields leading to partial body exposures. The gold standard, dicentric assay and other conventional methods have limited application in such scenarios. As an alternative, we propose Premature Chromosome Condensation combined with Fluorescent In-situ Hybridization (G_0_-PCC-FISH) as a promising tool for partial body exposure biodosimetry. In the present study, partial body exposures were simulated ex-vivo by mixing of uniformly exposed blood with unexposed blood in varying proportions. After G_0_-PCC-FISH, Dolphin’s approach with background correction was used to provide partial body exposure dose estimates and these were compared with those obtained from conventional dicentric assay and G_0_-PCC-Fragment assay (conventional G_0_-PCC). Dispersion analysis of aberrations from partial body exposures was carried out and compared with that of whole-body exposures. The latter was inferred from a multi-donor, wide dose range calibration curve, a-priori established for whole-body exposures. With the dispersion analysis, novel multi-parametric methodology for discerning the partial body exposure from whole body exposure and accurate dose estimation has been formulated and elucidated with the help of an example. Dose and proportion dependent reduction in sensitivity and dose estimation accuracy was observed for Dicentric assay, but not in the two PCC methods. G_0_-PCC-FISH was found to be most accurate for the dose estimation. G_0_-PCC-FISH has potential to overcome the shortcomings of current available methods and can provide rapid, accurate dose estimation of partial body and high dose accidental exposures. Biological dose estimation can be useful to predict progression of disease manifestation and can help in pre-planning of appropriate & timely medical intervention.

## Introduction

Increase in the use of ionizing radiation in medical, industrial and power sector has enhanced the need for emergency preparedness for management of possible accidental exposures, majority of which are likely to be gamma or x-ray exposures^1,2^. They maybe usually acute in nature and a combination of high dose and localized exposures, generally resulting in severe tissue injuries. On the other hand, near-whole-body exposures (WBE) result in acute radiation syndrome (ARS) or even mortality. The latent period between exposure and disease manifestation may be of the order of 1–4 weeks; hence, absence of initial symptoms and lack of accurate dose estimate may result in underestimation of severity of manifestation of symptoms, which are highly dose dependent. In most scenarios, dose may be estimated by physical reconstruction of events using physical parameters such as activity of source, distance, time spent, location of individuals, orientation and movement during the incident. However, in situations where enough data is not available for dose reconstruction, biodosimetry may help in predicting progression of ARS and in planning appropriate and timely medical interventions.

Chromosomal aberrations in peripheral blood lymphocytes are the most appropriate markers/indicators for radiation biodosimetry; dicentric assay particularly, has been universally accepted, gold standard method until date^3–7^. Conventionally, the aberration analysis is carried out on metaphases, after culturing the human blood lymphocytes for a minimum of 48–50 h. In high dose exposures, the availability of metaphases is reduced due to non-stimulation of cells, mitotic cell death or interphase cell death and most importantly, cell cycle arrests at checkpoints. In addition, severe leukopenia may set in within one to three days, reducing the lymphocyte counts in the blood sample.

To overcome the limitations of arresting of cell cycle check points at high dose exposures, Premature Chromosome Condensation (PCC) techniques were introduced for chromosomal aberration analysis. G_2_-PCC was introduced in 1996^8^ and later described by many more^9–12^. The assay allows relatively easy quantification of ring chromosomes, but not dicentrics. Although, rings are also radiation specific, their low yield is a limitation for partial body exposure (PBE) dosimetry. Furthermore, the method still requires 48 h of culture and scoring a large number of spread in case of localised exposure. In another approach, PCC is carried out in G_0_-phase (G_0_-PCC) lymphocytes by cell fusion with mitotic cells. It does not depend on cell cycle progression/metaphase availability and therefore, 48 h cell culturing step is eliminated. Aberration analysis can be performed within a few hours after blood collection in G_0_-phase.

The first reports for biodosimetry applications of G_0_-PCC were available in 1990s^13–16^ with a few studies citing its potential for non-uniform exposure dosimetry^17,18^. Conventionally, excess chromosomal bodies and rings were counted in non-fluorescent, Giemsa stained G_0_-PCC spreads. It comes with high background variation, and relatively difficult scoring. With the evolution of rapid fluorescent in-situ hybridization (FISH) techniques for identifying various types of aberrations, G_0_-PCC is currently being re-visited^19–28^. Chromosome painting with FISH allows more accurate detection and counting of exchanges, besides breaks. The technique is quick with ready to use probes with minimal or no pre-processing steps. A few studies have reported radiation response of specific chromosomes under uniform exposure conditions. Radiation response of chromosome 3 & 4, specific of aberrations up to 7 Gy, was reported earlier^29,30^. Reciprocal translocation frequencies in Chromosome 2 & 4, after 4 Gy gamma ray exposure, were found similar when counted with G_0_-PCC or G2-PCC or on metaphases^31^. Further, radiation response in the range of 0–6 Gy, of aberrations of chromosome 8, was also reported^32^. Another report studied chromosome 1, 2 & 4 specific aberrations after exposure to 2, 4 and 6 Gy of gamma radiation^33^. However, there are no reports of PBE biodosimetry using G_0_-PCC-FISH.

In the present study, we have evaluated G_0_-PCC-FISH as a rapid method for high dose biodosimetry for WBE as well as PBE with whole chromosome painting of Chromosomes 1, 2 & 4. As a pre-requisite, a broad dose range calibration curve was generated with human peripheral blood lymphocytes. Curve coefficients were estimated and validated from double blinded irradiated samples. Further, PBEs were simulated ex-vivo by mixing unexposed cells to the cells exposed to varying doses of gamma radiation. Sensitivity to detect cells from the exposed fraction, and accuracy in dose estimation were assessed. The outcomes are compared with conventional dicentric method and conventional Giemsa stained fragment counting in G_0_-PCC (G_0_-PCC-fragment). Comparative dispersion analysis of the aberrations for WBE and PBE was carried out to assess the potential of G_0_-PCC-FISH to differentiate between the two types of exposures. Finally, a methodology is proposed to discern if the received blood sample conforms to PBE or WBE and a multi-parametric dose estimation protocol is proposed accordingly. This will be helpful in practical situations of accidental exposures.

## Material and methods

### Reagents

Dulbecco’s Modified Eagle Medium (DMEM), Roswell Park Memorial, Institute Medium (RPMI1640), Fetal Bovine Serum (FBS) were purchased from Gibco, Phytohemagglutinin (PHA). Colcemid, Poly Ethylene Glycol (PEG, MW:1450 w/v 50%), Giemsa stain, Trypsin were purchased from Sigma, FISH probes for human whole chromosome painting were purchased from Metasystem probes. Methanol, Acetic Acid were purchased from Sisco Research laboratories Pvt. Ltd, and Potassium Chloride was purchased from Thomas Baker.

### Biological resources

Chinese Hamster Ovary (CHO) cells were procured from National Centre for Cell Science, a national institute, that serves as a cell repository and certified distributor for cell lines in the country.

Fresh human blood samples were collected with prior approval from Medical Ethics Committee, Bhabha Atomic Research Centre. All the methods were performed in accordance with the relevant guidelines and regulations of the ethics committee. Each of the donors were informed and written consent was obtained prior to blood collection. Blood was collected by a certified phlebotomist in lithium heparin coated tubes.

### Methodology

#### Uniform exposure studies: preparation and validation of calibration curve

Blood samples were collected from three healthy non-smoker, non-alcoholic volunteers, two males and one female (25–35Y), without any history of radiation exposure. Isolated PBMCs were irradiated to Co-60 Gamma radiation, in the dose range of 0–15 Gy, at dose rate of ~ 1 Gy/min, at the blood irradiator facility at Bhabha Atomic Research Centre. Calibrated radiation source was used with measurements traceable to the primary standard.

PBMCs were isolated using ficoll based density gradient medium. The cells were maintained in RPMI-1640 supplemented with 10% FBS without PHA stimulation, irradiated and incubated for 24 h at 37 °C in CO2 incubator. PBMCs were then mixed with mitotic Chinese Hamster Ovary (CHO) cells in a ratio of 5:1. The cells were centrifuged and cell fusion was carried out with the help of 50% w/v PEG (MW 1450,100 μl, 2 min). Post fusion, the cells were washed with RPMI-1640 and incubated for 2 h in PCC media (RPMI-1640 with 10% FBS, 1 μg/ml Colcemid). This was followed by a 6 min hypotonic treatment with KCl and fixation in 3:1 methanol-acetic acid. Detailed protocol for the same is reported previously^28^. Chromosomal spreads were prepared on a microscopic glass slide. Whole chromosome paint FISH was performed on freshly prepared slides as per manufacturer’s protocol provided along with the probes. Chromosome pairs 1, 2 & 4 were painted in red, green and yellow (red + green) respectively using fluorescent DNA probes. Imaging was carried out with Axio-imager microscope Z-2, using automated metaphase capture software Metafer-5 from Metasystems (Germany) utilizing DAPI, FITC and SpO excitation emission filters for FISH. For analysis, IKAROS and ISIS platforms of Metasystems were used.

Number of fluorescent signals were counted among spreads with distinct FISH signal for each chromosome pair. Each signal was confirmed with individual fluorescence signal and counterstain to exclude false positives. Fluorescent spots, excess to two, were counted as damage or excess signal which could either be generated from breaks or mis-joining of breaks (translocations). In total, from three donors, either ≥ 100 aberrations or ≥ 500 cells were counted for each dose point. Frequency of excess signals was plotted against dose to generate the calibration curve. For validation of the generated calibration curve, scoring of five dose blinded samples was carried out using G_0_-PCC-FISH and dicentric assay. For G_0_-PCC-FISH, aberrations in 50–100 cells were counted and dose estimations were carried out with the equation obtained from the generated calibration curve.

Dicentric assay was performed in the following manner. Irradiated blood samples were allowed to repair for 2 h at 37 °C in humidified, CO_2_ incubator. Subsequently, 0.5 ml blood was mixed with 4.5 ml RPMI medium supplemented with 10% FBS, and 10 μg/ml PHA. For metaphase arrest, long Colcemid treatment (0.02 μg/ml) was given, starting at 24 h till the end of the culture at 52 h. Metaphase harvesting was carried out as described previously (IAEA 2011). Either ≥ 500 cells or ≥ 100 dicentrics were counted in metaphase chromosomes for each sample. G2/M spreads without distinct centromere constrictions, where dicentrics were not clearly quantifiable, were ignored, irrespective of presence or absence of damage.

#### Simulated partial body exposure studies

**Figure 1:**
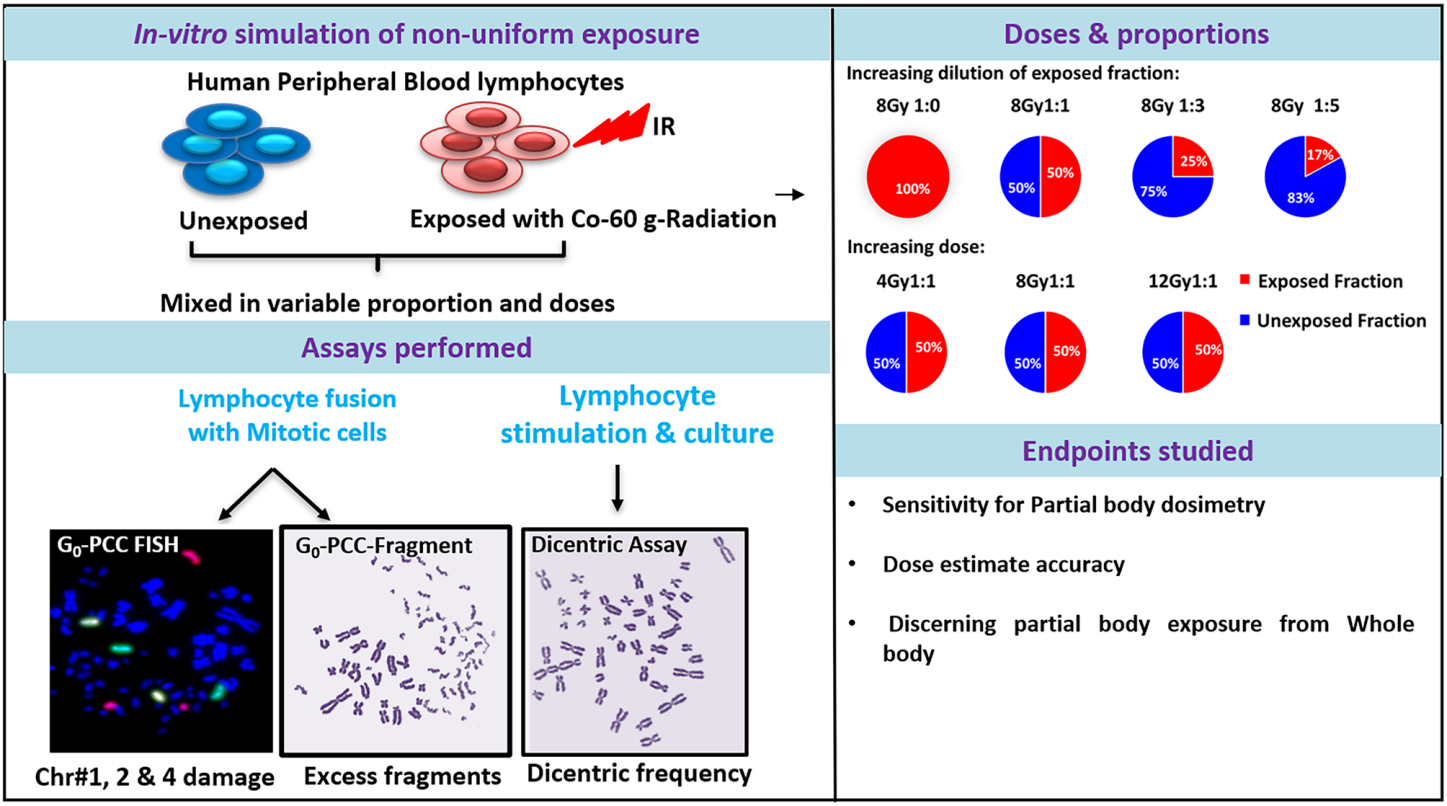
Methodology for partial body simulation study.

Towards assessment of G_0_-PCC for its potential application in partial body dosimetry, studies were conducted on ex-vivo irradiated and simulated PBEs. Blood samples were collected from three donors as described in previous section, irradiated to 4 Gy, 8 Gy and 12 Gy of Co-60 gamma radiation. The simulation of PBE was carried out by mixing of exposed blood with unexposed blood. Variable proportions as well as variable doses were studied to assess both dose and proportion dependent effect on sensitivity and accuracy of the methods. At a fixed dose of 8 Gy, the proportion of unexposed fraction to the exposed fraction was gradually increased from, 1:0 to 1:1 to 1:3 and finally 1:5. Additionally, doses were varied from 4 to 8 Gy and 12 Gy at a fixed proportion of 1:1. Dicentric and G_0_-PCC-FISH assays were performed as described in previous section and G_0_-PCC-Fragment assay was performed as reported earlier^27^. Either ≥ 500 cells or ≥ 100 dicentrics were counted in metaphase chromosomes for each sample. For G_0_-PCC-FISH, 50–100 spreads were counted and for G_0_-PCC-Fragment, on an average, 50 spreads were counted. Aberration frequency, distribution of aberrations, and proportions of un-aberrated and aberrated cells were extensively analyzed and compared with the corresponding uniform exposure. The detailed methodology is presented in Fig. 1**.** In total, 54 simulated samples; 6 sets, from each of the 3 donors using the 3 methods, were analyzed. With the obtained data, G_0_-PCC-FISH was evaluated for its sensitivity for PBE, accuracy in dose estimation and its ability to discern a PBE.

### Partial body dose estimation methodology

For dicentric assay, dose estimation was carried out using standard Dolphin’s method^4,34^. CABAS tool was used for DCA based dose estimation, wherever possible. Accordingly, yield of aberration, Y, among the exposed fraction is given by:

1 $$\frac{Y}{(1-{e}^{-Y})}=\frac{X}{\left(N-{n}_{0}\right)}$$

where;

*N* is the total number of cells scored, *X* is the sum of all observed aberrations and *n*_0_ is the total number of un-aberrated cells.

Extending the Dolphin’s method to G_0_-PCC assay, the expected yield was calculated by introducing the correction for background level of aberrated cells (*n*_bkg_). The Dolphin’s method assumes that all the aberrated cells and aberrations come from the exposed fraction. This assumption is valid for dicentrics, as background level of aberrated cells are rare. However, in G_0_-PCC methods, background correction is included.

Hence, Eq. (1) is modified for G_0_-PCC as follows:

2 $$\frac{Y}{(1-{e}^{-Y})}=\frac{X}{\left(N-{(n}_{0}+ {n}_{bkg)}\right)}$$

The Yield, *Y*, obtained using Eqs. (1) and (2) is used for estimating the dose (*D*), as discussed in the following sections.

## Results and discussions

### Uniform exposure studies

#### Calibration curve

**Figure 2:**
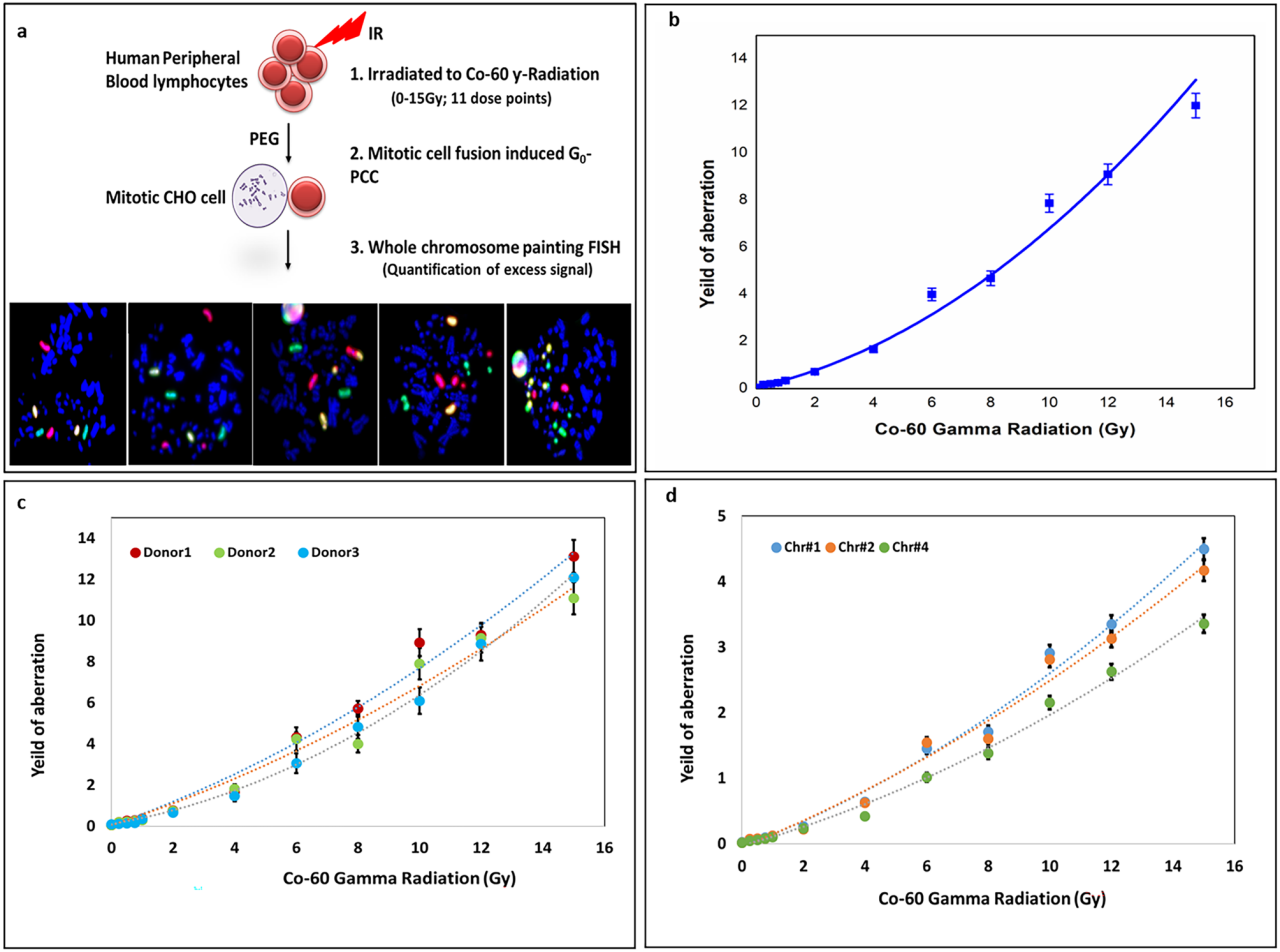
Ex-vivo radiation response of chromosome 1, 2 & 4 associated breaks & interchanges in human PBMCs assessed using G_0_-PCC-FISH. (**a**) Microscopic images of PCC spreads showing increasing number of aberrations with increasing dose (left to right). (**b**) Calibration curve from pooled data of aberrations of three donors. (**c**) Individual response curves of three donors and d) Individual response curves of the three chromosomes, pooled from three donors. The error bar represents mean ± Poisson Error.

Radiation response under uniform exposure was a pre-requisite for studying PBE. To obtain the same, calibration curve was generated for Co-60 gamma radiation in the dose range of 0–15 Gy, from three donors. Dose dependent increase in chromosomal aberrations was distinctly visible. Representative images of G_0_-PCC-FISH spreads with increasing frequency of aberrations are presented in Fig. 2a**.** The calibration curve from pooled data of the three donors is plotted in Fig. 2b while the individual data of the three donors is plotted in Fig. 2c. Further, radiation response for each of the chromosomes 1, 2 & 4 for each of the donors is plotted in Fig. 2d. The calibration curve follows a linear quadratic response, with the Yield, *Y*, being given as:

3 $$Y=\alpha D+\beta {D}^{2}+c$$

where *α* = 0.28 ± 0.037 Gy^-1^, β  = 0.039 ± 0.004 Gy^-2^ and *c* = 0.0568 respectively (R^2^ = 0.985).

Response of aberrations in each donor and each of the chromosomes is also found to be linear quadratic, with negligible inter-individual variations. Overall aberrations combined for all doses, were highest for chromosome 1, followed by that for chromosomes 2 & 4 respectively. The frequencies of aberrations in chromosomes 1 & 2 were not statistically significant, although, the response of chromosome 4 was significantly lower than the other two chromosomes at multiple dose points, particularly beyond 2 Gy. Chromosome dependent responses are largely attributed to the genome size of the chromosomes. Similar results were reported earlier for metaphase based translocation assay^35^. Linear quadratic response for breaks and interchanges has been reported for chromosomes 3 and 4 using G_0_-PCC-FISH^29^, wherein the response was generated 8 h after irradiation to doses 1, 3, 5 & 7 Gy of gamma radiation. Another study has reported linear quadratic response in human chromosome no. 8 using G_0_-PCC-FISH in chromosome^32^. A study limited to doses 2, 4 & 6 Gy for Chromosomes 1, 2 & 4 reported similar radiation responses^33^. In constrast to our data, one study has reported linear response for chromosomes 1 & 2 in the dose range 0–5 Gy using chemically induced G_0_-PCC-FISH^36^. In this study, relatively higher aberration yield for chromosome 2 than for chromosome 1 was also reported. The differential sensitivity between two chromosomes was not consistent in our data. The disagreement in the observation could be attributed to (1) difference in the degree of chromosome condensation between chemically vs. cell fusion induced G_0_-PCC, (2) scoring of limited number of cells from single individual and (3) limited dose range.

In G_0_-PCC-FISH, any number and combinations of chromosomes can be painted with chromosome specific color probes. The most popular ones are; one color, two color, three-color and Multi-plex FISH, which paint one, two, three chromosomes and entire genome (24 colors with 22 autosomes, X and Y) respectively. In this study, three color FISH has been carried out. Two, major considerations for the choice are; processing duration of FISH and sensitivity of the assay. Three color FISH requires same duration as one- or two-color FISH (< 24 h), and has more sensitivity (aberration/spread) due to higher genome proportion coverage (~ 22% compared to ≤ 8% and ≤ 16% in one- and two-color FISH respectively). With its higher sensitivity, it requires lesser number of spreads to be scored for dose estimation. While Multiplex FISH can be more sensitive needing only small number of spreads to be scored (20–25 for a uniform exposure), it demands longer duration (~ 48 h) for FISH processing, thus defeating the purpose of rapid biodosimetry. It is also likely that, for a localized exposure, counting < 50–100 cells may result in false negative dose estimation or incorrect dose, thereby not serving the purpose of scoring lesser number of cells either. Hence, three-color FISH appeared as a balanced option.

It is worth emphasizing here that this is the first study on G_0_-PCC-FISH with multi-donor, broad range exhaustive calibration curve, enabling a robust statistical distribution of aberrations and accurate estimates of curve coefficients. Further, extensive dispersion analysis of aberrations from this data were performed which helped in identifying parameters to discern PBE from WBE. These results are discussed in a later section.

#### Validation of calibration curve

For validation of the calibration curve, dose estimations of five double blinded samples were carried out and the estimated doses were compared with those obtained from conventional dicentric assay (Table 1). Yield of aberrations (*Y*) was calculated from both the methods and dose was estimated for G_0_-PCC-FISH using Eq. 3, whereas for dose estimation with dicentrics, published curve equation (Eq. 4)^37^ was used:

**Table 1:** Comparison of true dose and the doses estimated using conventional dicentric assay and G_0_-PCC-FISH assay.

True dose (Gy)	G_0_-PCC FISH Chr #1, 2 & 4 (~ 50–100 cells per sample)		Dicentric Assay (100 dicentrics or ≥ 500 cells per sample)	
	Estimated Dose [LL, UL] (Gy)	Error (%)	Estimated Dose [LL, UL] (Gy)	Error (%)
0	0.2 [-0.1, 0.4]	< MDL	0 [0, 0]	< MDL
1	1.2 [0.8, 1.5]	20%	0.8 [0.6, 1]	20%
2	2.2 [1.6, 2.8]	10%	1.7 [1.5, 1.9]	15%
4	4.4 [4.0, 4.7]	10%	3.5 [3.2, 3.9]	12%
8	7.5 [6.8, 8.1]	6.2%	7.4 [6.7, 8.1]	7.3%

Values in the bracket represent estimates of lower limit (LL) and upper limit (UL) at 95% confidence and Error represents % deviation from the true dose.

4 $$Y=0.027D+0.065 {D}^{2}+0.0005$$

The G_0_-PCC-FISH method was found to be as accurate as dicentric assay in the tested range of 1- 8 Gy, with all the estimates lying within 20% of the true value. Error in the dose estimates reduced with increasing dose. At lower doses, aberrations are relatively lower and contribution by background and individual response variations can result in higher errors compared to that at higher doses.

### Simulated partial body exposure studies

#### Sensitivity of G_0_-PCC-FISH for partial body exposure

**Figure 3:**
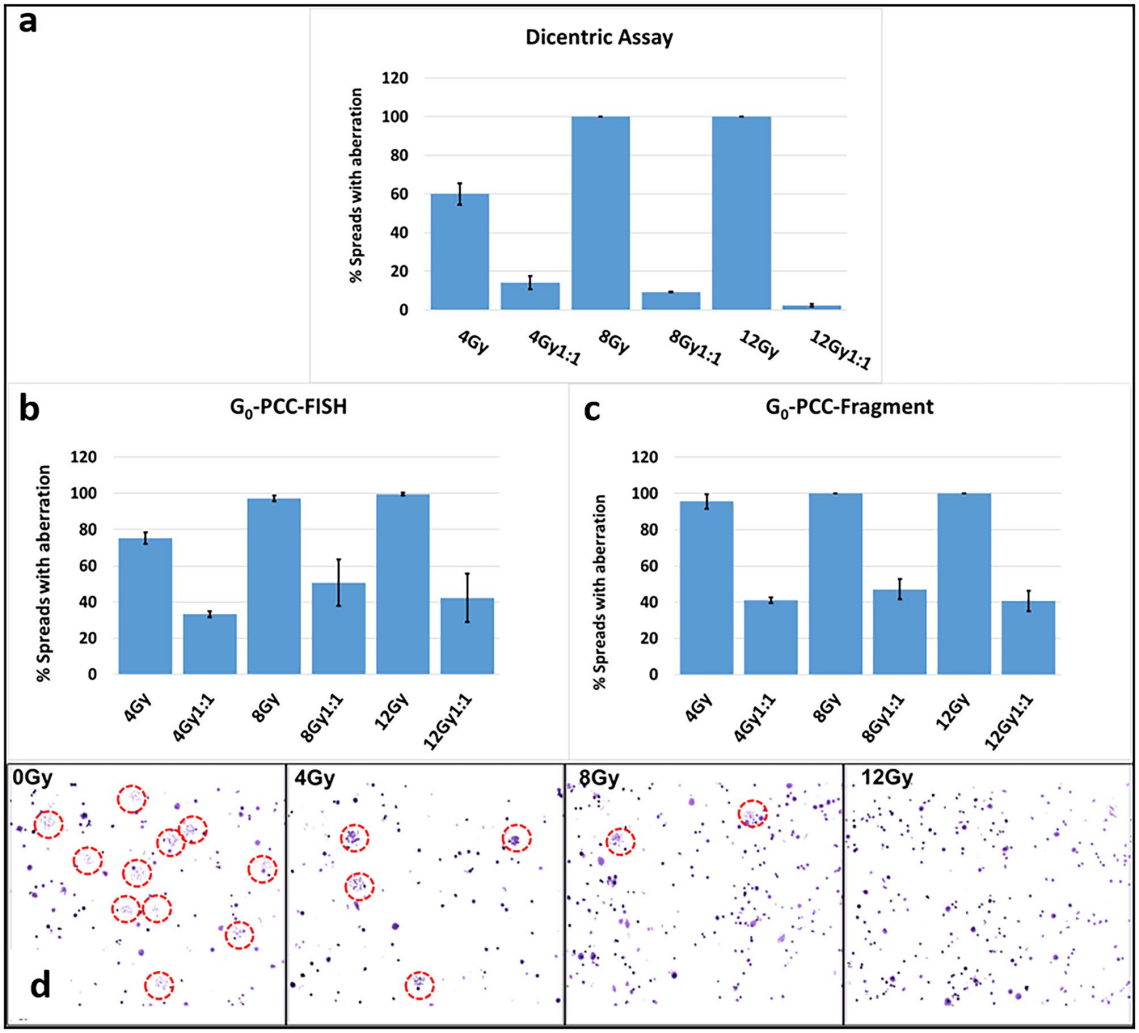
Sensitivity of different methods for detection of aberrant cells from the exposed fraction in PBE relative to WBE of the same dose in *in-vitro* simulated exposures using human blood lymphocytes. The proportion 1:1 refers to samples with 50% exposed proportion. (**a**) dicentric assay (**b**) G_0_-PCC-Fragments (**c**) G_0_-PCC-FISH. (**d**) Typical 10 X fields of metaphase preparation in dicentric assay with increasing uniform exposure. Metaphases are encircled in red boundaries. For both the PCC methods, data was corrected for background level of aberrated cells in case of PBE. Data bars represent mean ± SD.

Sensitivity of a method for PBE refers to efficiency to detect cells from the exposed fraction from the mixed pool. While un-aberrated cells may come from both exposed as well as un-exposed fraction, aberrated cells majorly represent the exposed fraction. Hence to evaluate the sensitivity of the three methods viz., dicentric assay, G_0_-PCC-Fragment and G_0_-PCC-FISH assay, we estimated proportion of aberrated cells in case of partial exposure (50%, 4 Gy, 8 Gy and 12 Gy) and plotted with that of corresponding uniform WBE of the same dose (Fig. 3**)**.

It was observed that in case of dicentrics, sensitivity to detect aberrated cells from the exposed fraction was lower relative to the two G_0_-PCC methods. For instance, 4 Gy (WBE) sample has shown 60% ± 5.2 cells having dicentrics whereas in 50% exposed sample, only ~ 15% cells are found to have dicentrics instead of the expected ~ 30% (Fig. 3a**)**. Efficiency has further declined with increasing doses; in case of 8 Gy and 12 Gy uniformly exposed samples, 100% cells are found to be aberrated whereas 50% exposed samples showed only < 10% and < 5% spreads having dicentrics. Further, in case of both G_0_-PCC methods, the proportion of cells with aberrations were in accordance with the proportion of exposed cells within ± 10% error (Fig. 3b, c). The lower sensitivity of dicentric assay can be explained on the basis of dose dependent decrease in cell division rate of exposed fraction and apoptosis in interphase, leading to a reduction in metaphase index (Fig. 3d). Hence, in such cases, while using conventional methods specialized equipment with automated high throughput imaging and scoring may be required to analyze huge number of cells^6,38–40^. However, owing to higher sensitivity, G_0_-PCC has the potential to overcome this shortcoming of conventional assay with scoring of 50–100 spreads, which, can be easily scored in a simple, manual fluorescent microscope as well.

#### Dose estimations

T_O_ evaluate accuracy of this method in partial body dosimetry, dose estimations from dicentric, G_0_-PCC-FISH and G_0_-PCC-Fragment assays were carried out and error in dose estimates were also calculated. For dose estimation, yields of aberration in the exposed fraction were calculated as described in the methodology section using Eqs. (1) and (2) and were subsequently used for calculation of corresponding dose estimates using appropriate curve equations. For G_0_-PCC-FISH and dicentric assay Eqs. (3) and (4) were used, whereas, for G_0_-PCC-Fragments the following equation, from a previously reported calibration curve^27^ was used:

5 $$Y=1.09 D+0.19$$

**Figure 4:**
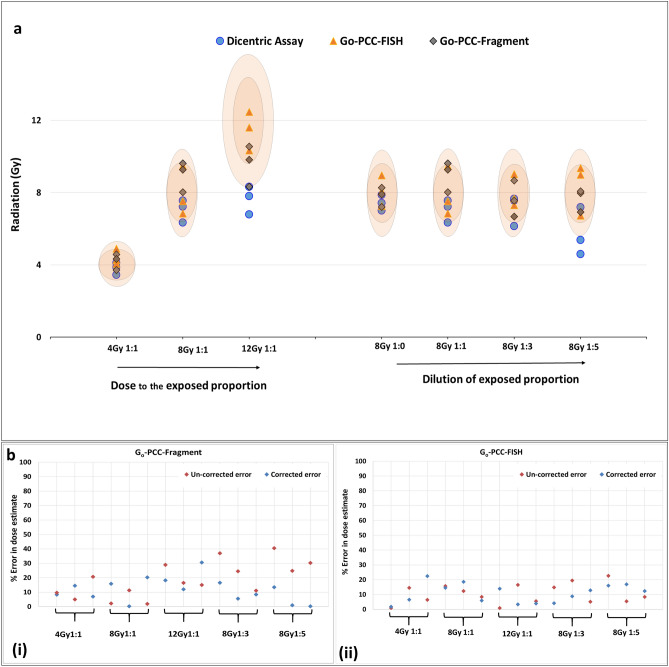
Dose estimates among simulated PBE samples (**a**) Dose estimates of three different donors for all the three methods. 1:0, 1:1, 1:3, 1:5 refers to ratio of exposed vs. unexposed fraction of the blood. Small and bigger orange ellipticals refer to 20% and 30% error vertically. Doses were heavily underestimated in case of dicentric assay at 12 Gy, 1:1 in all the three donors and at 8 Gy, 1:5 in two of the three donors. (**b**) Impact of background correction in dose estimation using G_0_-PCC- methods. (i) G_0_-PCC-FISH (ii) G_0_-PCC-Fragments. While there is no significant impact of background correction in G_0_-PCC-FISH, dose estimation error is significantly reduced in G_0_-PCC-Fragments, specially at higher unexposed proportions.

Obtained estimates for each donor, with each method are plotted in Fig. 4a**.**

In case of dicentric assay, severe underestimation (> 30% error) was observed at 12 Gy and 8 Gy, 1:5 proportion. Nevertheless, for 0, 1:1 & 1:3 proportion, dose estimates were close to true dose with scoring of ≤ 500 cells. Hence, it can be concluded that with increasing dose and decreasing exposed fraction, sensitivity of dicentric assay decreases and accuracy of dose estimate gets compromised.

Dose estimates in both G_0_-PCC-Fragments and G_0_-PCC-FISH did not suffer dose or proportion dependent underestimation. G_0_-PCC-FISH is found to be more accurate than G_0_-PCC-Fragment based dose estimation and background correction was needed to reduce the error at 1:5 proportion. In case of G_0_-PCC-FISH, all the dose estimates, except one, were within ± 20% error, with or without background correction. Further, with G_0_-PCC methods, due to high sensitivity, substantially low cell counts are sufficient. Hence, it can be inferred that G_0_-PCC methods are more suitable for high dose, PBE dosimetry, with the added advantage of being rapid. Specifically, G_0_-PCC-FISH estimated the doses with higher accuracy compared to G_0_-PCC-Fragment based technique. It is worth mentioning that while the calibration curves for G_0_-PCC-Fragments and G_0_-PCC-FISH are established up to 15 Gy, Dicentric assay is only up to 6 Gy. This raises a doubt whether the calibration curve could have contributed in underestimation of the doses in dicentric assay. The experimental results indicate that calibration curve holds good till 8 Gy although metaphase index may be lower; for 8 Gy dose point, as the proportion of unexposed fraction increased, accuracy of dose estimation was compromised, nevertheless, accurate estimates were obtained for uniform and 1:1 proportion. As anticipated, at 12 Gy, for uniform exposures, ~ 20% underestimation was observed while for PBE cases it was > 30%. In such a case, underestimation can be partly attributed to the calibration curve and partly to lower metaphase index from the exposed fraction.

Furthermore, in the methodology, we used background correction for both the PCC methods (Eq. 2) with the assumption that aberrated cell proportion also contains background level of aberrated cells from the unexposed fraction. When, unexposed fraction is large, the contribution of these cells resulted in considerable underestimation of doses at higher unexposed fractions, especially in G_0_-PCC-Fragment assay.

For G_0_-PCC fragment background level of aberration is ~ 20 per 100 cells (Eq. 5), corresponding to 1 aberration for every 4 un-aberrated cells (n_0_). Similarly, for G_0_-PCC-FISH considering the background level of aberration of 0.057 cells (c in Eq. 3), 6 aberrations per 100 n_0_ were estimated as background.

Accordingly, for every background count, one single aberrated cell (n_1_) was considered as background (n_bkg_) and added to the cells with n_0_. The associated aberrations were also subtracted from total aberrations. Cells with more than 1 aberrations were counted as background when n_1_ were inadequate. It is noteworthy that, for low dose exposures the un-aberrated cells are contributed from exposed fraction as well. Hence, background correction should be done only after ascertaining PBE of doses ≥ 4 Gy.

To demonstrate utility of the background correction, % errors in dose estimates were plotted with or without background correction as shown in Fig. 4b. Background correction was more impactful in keeping the errors within 30% in G_0_-PCC-Fragment method, especially where un-exposed fractions were higher (8 Gy, 1:5) as observed in Fig. 4b(i). This can be attributed to higher background aberration frequency in unexposed fraction in G_0_-PCC-fragment compared to that in G_0_-PCC-FISH. G_0_-PCC-FISH practically did not benefit from background correction in our study, where the probability of contribution of background is very low at all proportions (Fig. 4b(ii)). Error remained within 20% for all the dose estimates except one in both corrected as well as un-corrected estimates. However, the equation has been maintained with background correction so it remains independent of the exposed fraction and can be used for both the PCC methods.

It is noteworthy that all G0-PCC assays have been conducted 24 h after exposure with scientific and practical reasons. Previously it was reported that optimal duration between exposure and processing or collection of samples is about 8–24 h based on chromosomal break repair kinetics^28^. In addition, for a PBE, lymphocyte pool recirculation time also needs to be considered. One circulation of peripheral blood takes only < 1 min to travel from and back to the heart. Hence, for a localized exposure, lasting for more than couple of minutes, immediately collected blood will not represent true PBE. However, only about 2% of lymphocytes are present in the peripheral blood while the remaining are present in lymphoid pool, which is relatively stagnant. Equilibration of lymphocytes between these two pools requires a duration of about 8–12 h, and the blood can be collected following this period. Furthermore, considering the delays attributed to detection of incident, reporting, logistic responses and transporting the exposed individual or reaching the site for collection of samples, it is also a most practical time window wherein the sample can be collected by biodosimetry team or medical professionals.

#### Dose estimation for PBE based on the discerning key parameters

**Figure 5:**
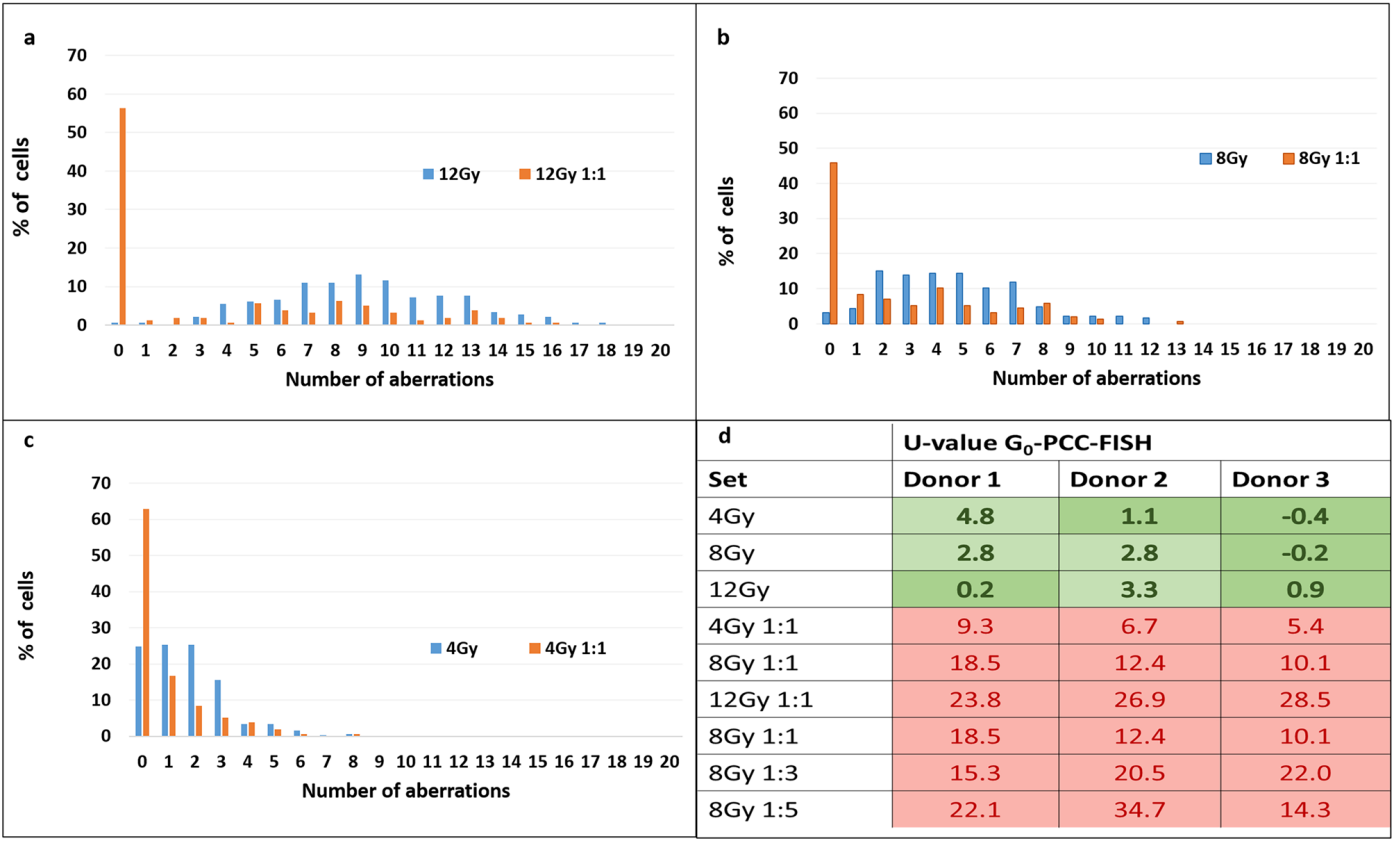
Distribution of aberrations for a given dose with WBE and after 1:1 mixing with unexposed cells (50% PBE). (**a**) 4 Gy, (**b**) 8 Gy, (**c**) 12 Gy andd) Quantification of dispersion with U-test among three donors. U-value within ± 1.96 refers to Poisson Distribution (dark green cells) greater value refers to greater deviation from Poisson (red cells).

Differentiation of PBE from WBE is very crucial for dose estimation. For this, we compared the dispersion of aberrations among WBE and PBE of the same dose and their deviations from Poisson were quantified by Papworth’s U-test (Fig. 5a, b, c). On applying Papworth’s U-test, it was observed that, in case of WBE, though overdispersion was observed, the distribution was closer to Poisson whereas, in all the PBE samples, considerable deviation from Poisson was observed (U-value range: 5.4–32.9). Deviations in WBE were moderate and hence the Dolphin’s method was able to provide dose estimates within reasonable uncertainty. U-test has been considered to be a good indicator of PBE in case of dicentrics. However, as observed in Fig. 5**,** signals counted in G_0_-PCC may not strictly follow Poisson distribution and large overdispersions may be observed at lower doses. As a result, U-value can only be indicative of PBE. Therefore, instead of exclusive reliance on U-test, a multi-parametric approach should be considered when using G_0_-PCC for discerning PBE.

**Figure 6:**
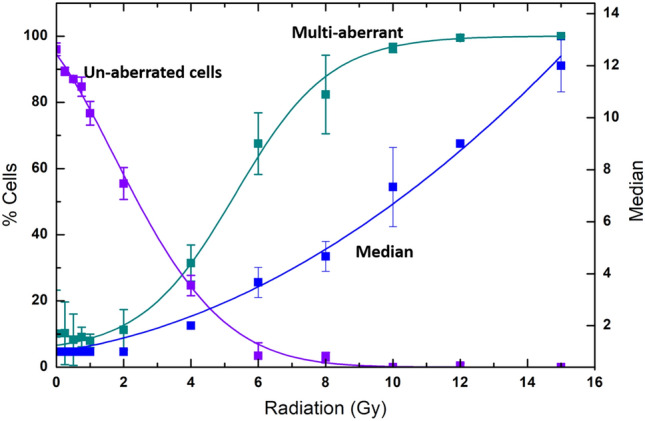
Radiation response of the key parameters under WBE condition: (i) *P1*, Median of aberrations among aberrated cells shows a linear quadratic response (ii) *P2*, proportion of multi-aberrant cells (X ≥ 3) among aberrated cells shows a sigmoidal response and (iii) P3, proportion of un-aberrated cells among total cell population depicts an exponential decrease.

After comparing the distribution of aberrations among WBE and PBE, we could identify three key parameters that can be used in combination to differentiate the two types of exposures for a given yield of aberrations; (i) median of aberrations among aberrated cells (*P1*), (ii) proportion of multi-aberrant cells (with ≥ 3 aberrations) among aberrated cells (*P2*) and (iii) proportion of un-aberrated cells among total scored cells (*P3)*. The first two are dependent more on the dose to the exposed fraction and remain largely un-affected by the amount of exposed fraction. The three parameters show a specific response under uniform exposure which is plotted in Fig. 6**.** For a given whole body dose estimate (D_WBE_), substantial deviation from the expected response of key parameters can confirm non-uniformity. Figure 6 shows that P1 was 1 for doses ≤ 2 Gy, and further increased in a linear quadratic manner. On the other hand, *P2* follows a sigmoidal pattern wherein, up to 2 Gy, majority of the aberrations are contributed by single aberrated cells; while beyond 2 Gy, multi-aberrant cells increase exponentially up to 8 Gy before saturating to ~ 100%. In contrast, *P3 is* > 90% for doses below 1 Gy, and decreases exponentially with increasing dose.

**Figure 7:**
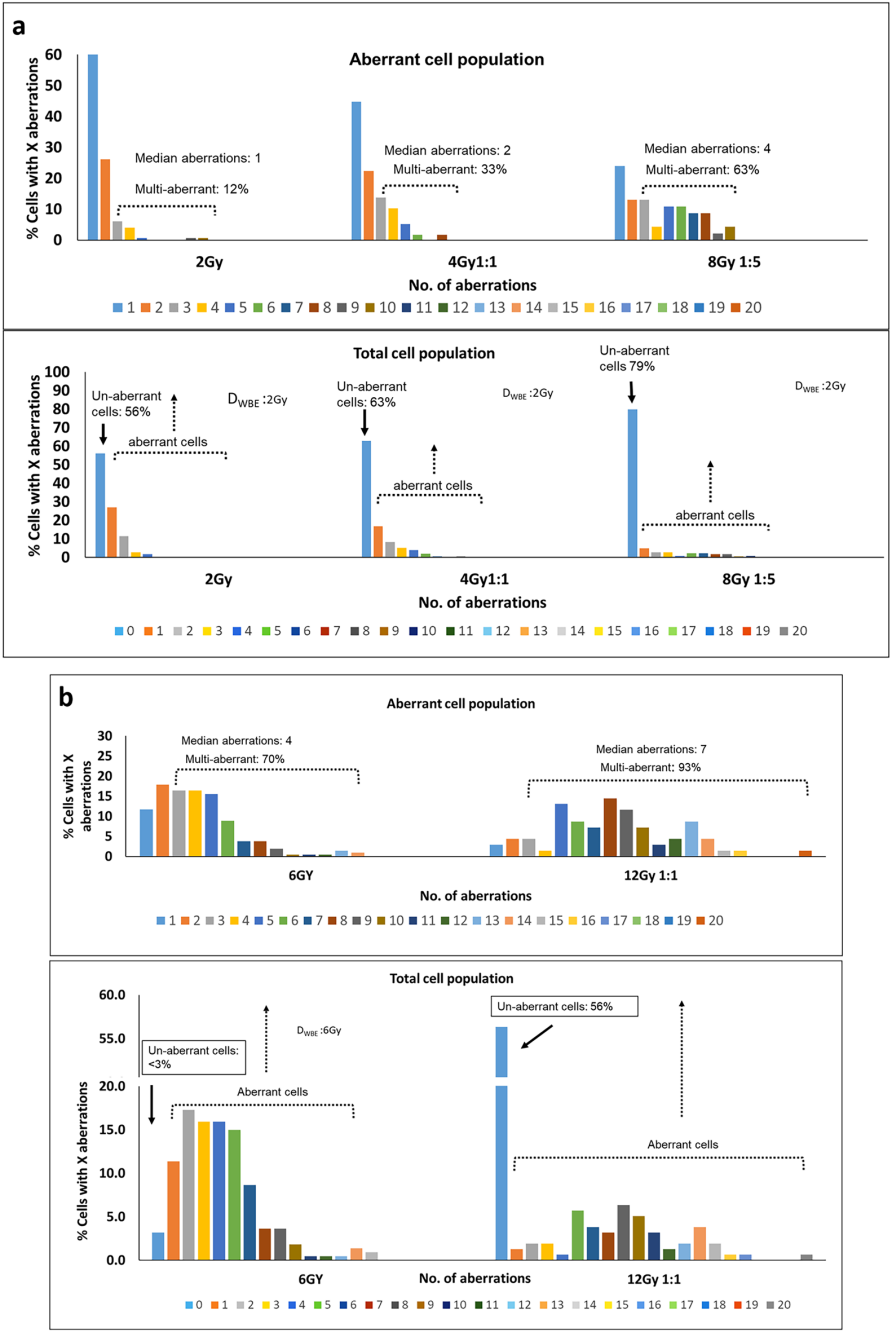
Distribution of aberration among irradiated lymphocytes after WBE and PBE with similar dose estimate in G_0_-PCC-FISH. (**a**). Samples with ~ 2 Gy D_WBE_; arising from 2 Gy WBE, or from 4 Gy, 1:1 and 8 Gy, 1:5 PBE. (**b**) and in samples with ~ 6 Gy D_WBE_ arising from WBE 6 Gy exposure or 12 Gy 1:1 PBE.

In Fig. 7, we have demonstrated that different WBE and PBE samples, with different exposure scenarios, can result in similar yield leading to similar D_WBE_; nevertheless they can still be differentiated based on the key parameteres. For example, D_WBE_ of 2 ± 0.1 Gy is possible under any one of the following three scenarios, (i) 2 Gy WBE, (ii) 4 Gy 1:1 PBE and (iii) 8 Gy 1:5 PBE. For this example, the total cell population and aberrant cell population, along with the key parameters are plotted in Fig. 7a**.** As may be seen from the aberrant cell population, the *P1*is 1, 2 and 4 for 2 Gy and 4 Gy 1:1 and 8 Gy 1:5 respectively while the corresponding *P2* values are 10%, 33% and 63%. The dose estimates corresponding to these two paramters (see Fig. 6) are ≤ 2 Gy, 3.3–4.0 Gy and 6.8–7.0 Gy respectively. On the other hand, the data from total cell population shows unexpectedly high value of *P3* which is also indicative of PBE. Similarly, in Fig.  7b, 6 Gy WBE and 12 Gy 1:1 PBE yields give rise to similar dose estimates but difference in the three key parameters again confirms the non-uniformity of exposure in the latter case. Hence, with the above example, it can be concluded that key parameters have potential to distinguish a partial exposure from a uniform exposure.

It is noteworthy that besides discerning the nature of exposure, median and % multi-aberrant cells can also be used for dose estimation. Once, PBE is confirmed, dataset can be corrected for background for further dose estimation with Dolphin’s approach. Also, *P1* and *P2* from the corrected dataset can provide more accurate dose estimation.

A step by step methology for discerning the type of exposure and dose estimation of an unknown sample is disscused in the next section.

#### Stepwise protocol for partial body dosimetry of an unknown sample post G_0_-PCC-FISH: an example

*Sample preparation:* Blood sample with unknown dose is collected. PBMCs are isolated and fusion with mitotic cells is performed. 2 h post fusion, cells are harvested, treated with hypotonic, and fixative. Slides are prepared and FISH is performed. Excess fluorescence signals are counted. For details of G_0_-PCC-FISH refer to methodology section and^28^.

Example dataset:

Assume that the scoring of aberration results in the following dataset:

**Table:** 

n_0_	n_1_	n_2_	n_3_	n_4_	n_5_	n_6_	n_7_	n_8_	n_9_	n_10_	X	Ν
182	11	6	6	2	5	5	4	4	1	2	193	228

Where n_0_, n_1_… refer to cells with 0, 1 … aberrations, X refers to total number of aberrations and *N* is total number of cells.

*Initial dose estimation:* Yield, *Y* is calculated as X/N. Assuming WBE and the dose estimate (D_WBE_) of is obtained by solving Eq. (3**).**

D_WBE_ = 2 Gy.

*Decision on WBE/PBE:* All the key parameters expected from 2 Gy WBE were deduced from Fig. 6. Observed values of key parameters were extracted from the scored data. The doses corresponding to the observed values were estimated from standard curves in Fig. 6**.** Disagreement between observed and expected values was an indication of PBE as presented below:

**Table:** 

Key parameters	Expected	Observed (D_WBE_ : 2 Gy)	Dose corresponding to observed values	WBE/PBE
1. P1	1	4	6.8 Gy	PBE
2. P2	10%	63%	6 Gy	PBE
3. P3	62%	79%	NA	PBE

*PBE dose estimation:* Once PBE is confirmed, dose estimation is performed with background correction. Background aberrations were calculated as 6% of n_0_ hence, 11 single aberrated cells from *n*_1_ population were considered as background (n_bkg_). These cells are added with n_0_ cells and their aberrations subtracted from total aberrations (corrected X = 182, N − (n_0_ + n_bkg_) = 35). Further, corrected *P1* and *P2* were calculated to be 5 and 78.3% respectively. Yield of the exposed fraction was calculated from Dolphin’s approach by solving Eq. 2 & dose estimates were obtained using Eq. 3, *P1* and *P2* as shown below:

1. Dolphin’s approach: D = 8.4 Gy
2. Using *P1*: D = 8.2 Gy
3. Using *P2*: D = 7 Gy

## Conclusions

Dosimetry of high dose radiation exposure is necessary to predict progression of disease manifestation and can help in pre-planning of appropriate & timely medical intervention. It is likely that high dose accidental exposure is partial body in nature. The gold standard biodosimetry method, dicentric assay has limited applicability at high doses. It tends to underestimate the dose with increasing extent of non-uniformity as well as dose to the exposed fraction. Moreover, it requires 48–52 h of culture, needs large number of cells to be scored and hence, may effectively need 3–5 days for dose estimation. In the present study, it is demonstrated that G_0_-PCC offers higher efficacy and sensitivity to detect PBE at high doses. In comparison, 500–> 1000 metaphases need to be analyzed in dicentric assay, 50–100 PCC spreads were found to be sufficient for dose estimation at all the simulated non-uniform exposures. The limitation of dose and non-uniformity dependent underestimations are not observed for G_0_-PCC in the tested range. G_0_-PCC based chromosomal aberration analysis is not subjected to cell cycle arrest and no cell culture setup is required, hence, dose estimates can be obtained in less than 24 h. G_0_-PCC-FISH is found to be more accurate than G_0_-PCC-Fragment. Three key parameters were identified which can discern a PBE and can also provide a close dose estimate. In conclusion, G_0_-PCC-FISH is rapid, sensitive method which can distinguish a PBE from uniform WBE and provide multi-parametric accurate dose estimation. Limitations of the method include the need of mitotic cells and optimized cell fusion protocols. The study has been carried out with homogeneous gamma radiation to the exposed part however, accidental conditions can be complex including High LET exposures, mixed radiation type of exposures or protracted exposures for which limitations and advantages of this method need to be explored. Further, dose estimations of in-vivo samples should be carried out for further validation of the methodology.

## Data Availability

The datasets generated during and/or analysed during the current study are available from the corresponding author on reasonable request.
